# A novel STAT3 inhibitor attenuates angiotensin II-induced abdominal aortic aneurysm progression in mice through modulating vascular inflammation and autophagy

**DOI:** 10.1038/s41419-020-2326-2

**Published:** 2020-02-18

**Authors:** Qi-ying Wu, Zhao Cheng, Yang-zhao Zhou, Yuan Zhao, Jian-ming Li, Xin-min Zhou, Hong-ling Peng, Guang-sheng Zhang, Xiao-bo Liao, Xian-ming Fu

**Affiliations:** 1 Department of Cardiovascular Surgery, The Second Xiang-ya Hospital, Central South University, Changsha, Hunan P.R. China; 2 Department of Hematology, The Second Xiang-ya Hospital, Central South University, Changsha, Hunan P.R. China

**Keywords:** Chronic inflammation, Aortic diseases

## Abstract

Abdominal Aortic aneurysm (AAA) is associated with chronic inflammation, cells apoptosis, and impairment of autophagy. BP-1-102, a novel potent STAT3 inhibitor, has been recently reported to significantly block inflammation-related signaling pathways of JAK2/STAT3 and NF-κB, as well as regulate autophagy. However, its role in vascular inflammation and AAA progression remains to be elucidated. In the present study, the effect and potential mechanisms of BP-1-102 on angiotensin II (AngII) induced AAA in ApoE^−/−^ mice were investigated. AAA was induced in ApoE^−/−^ mice with infusion of AngII for 28 days. BP-1-102 was administrated orally to mice every other day. Mice were sacrificed on day 7, day 14, and day 28 to evaluate the treatment effects. BP-1-102 markedly decreased AAA incidence and aortic diameter, maintained elastin structure and volume, reduced the expression of pro-inflammatory cytokines and MMPs, and inhibited inflammatory cells infiltration. Moreover, BP-1-102 dramatically reduced the expression of JAK2, p-STAT3, p-NF-κB, and Bcl-xL but maintained the expression of LC3B and Beclin in AAA tissues. In vitro, vascular smooth muscle cells (VSMCs) were treated with AngII and/or BP-1-102 at indicated time and concentration. BP-1-102 inhibited AngII-induced JAK2/STAT3 and NF-κB signaling activation and maintained autophagy-related proteins expression in VSMCs. Taken together, our findings suggest that BP-1-102 inhibits vascular inflammation and AAA progression through decreasing JAK2/STAT3 and NF-κB activation and maintaining autophagy.

## Introduction

Abdominal aortic aneurysm (AAA) is defined as a focal dilation of the aortic wall which is associated with aortic rupture. It affects up to 8% of men over the age of 65 years in the United States, with an annual mortality of more than 15,000^1,2^. As the elderly population increases, AAA becomes a more serious health problem. The current treatment of patients with a large AAA includes elective surgical or endovascular repair to prevent rupture. Many patients with a small AAA are detected during routine screening; however, there is no effective therapeutic option to prevent the progression of AAA. For treating small AAA, many researchers are seeking novel therapeutic approaches, including pharmacological therapy and cell transplantation therapy^3,4^. Their efficacy on AAA formation had been confirmed in experimental studies. However, there is no evidence for beneficial in clinical trials^5^. Therefore, the treatment of small AAA remains an imperative clinical problem^6^.

Chronic inflammation, vascular smooth muscle cells (VSMCs) apoptosis and extracellular matrix (ECM) degradation are the main pathological characteristics of AAA^7^. Previous studies have highlighted the significance of vascular inflammation in the degradation and remodeling of ECM, and its key role in the pathogenesis of AAA. Inflammatory cells secrete proteolytic enzymes, including matrix metalloproteinases (MMPs) that are involved in the degradation of ECM in aortic walls. Much evidence has confirmed that Janus kinase/signal transducers and activators of transcription (JAK/STAT) and nuclear factor (NF)-κB as key signal integrators controlling the process of vascular inflammation and AAA formation. For example, metformin has been reported to inhibit the angiotensin II (AngII) induced AAA progression in mice through decreased the activity of NF-κB and STAT3 signal pathway^8,9^. Embelin inhibits AAA formation through decreasing IL-6 induced STAT3 and NF-κB inactivation^10^. NF-κB is a family of transcriptional factors implicated in the processes of both inflammatory responses and oxidative stress which can promote chronic inflammation in the aortic wall, and regulate MMPs transcription^11^. NF-κB activities are elevated in human AAA tissues as well as experimental AAA^12^. Previous studies reveal that AngII-induced NF-κB activation plays a central role in the development of AAA through regulation of gene expression of inflammatory molecules^13^. Moreover, NF-κB inhibitor has been shown to significantly inhibit the formation of AAA in animal model^14^. JAK2/STAT3 signaling pathway is another important pathway mediating vascular inflammation during AAA progression. Activation of different JAK2/STAT3 pathway components, including p-STAT3 and t-STAT3 has been observed in AAA tissues. A more recent study has found that AngII-induced AAA formation in ApoE^−/−^ mice depended at least in part on the AngII-mediated activation of STAT3^15^. Furthermore, a STAT3 inhibitor (S3I-301) could reduce the incidence and severity of AngII-induced AAA formation and decrease MMPs activity and the ratio of M1/M2 macrophages^16^. These results suggest that JAK2/STAT3 and NF-κB signaling pathways may be potential therapeutic targets for treatment of AAA.

Autophagy is a reparative, life-sustaining process in eukaryotic cells, by which cytoplasmic components are sequestered in double-membrane vesicles and degraded on fusion with lysosomal compartments^17^. Growing evidence reveals that basal autophagy is an essential in vivo process mediating proper vascular function^18,19^. Microarray analysis of AAA tissues reveals upregulation of several autophagy-related genes (LC3, and BECLIN1) in AAA tissue suggesting induction of autophagy^20^. Several previous studies highlight the protective role of autophagy in mitigating the AngII-vascular inflammation and AAA formation^19–21^. Loss of autophagy in VSMCs promotes VSMCs death and endoplasmic reticulum stress-dependent vascular inflammation and aggravates AAA^19^. These evidences indicate that autophagy is protective for AAA formation.

BP-1-102 is a novel small molecular STAT3 inhibitor with a high affinity. The application of STAT3 inhibitor in cancer therapy has been widely studied^22^. Previous study has found that a STAT3 inhibitor, S3I-301 decreased the incidence and severity of AngII-induced AAA formation and decreased MMPs activity and the ratio of M1/M2 macrophages in AAA tissue^16^. It is reported that S3I-201 is neither effective at low concentrations nor orally bioavailable. BP-1-102 is an analog of S3I-201 and designed as a new orally bioavailable STAT3 inhibitor that functions by directly interacting with STAT3 at a relatively low concentration which is a notable improvement. It is proved that BP-1-102 not only blocks the pathways of JAK2/STAT3 and NF-κB, but also targets Bcl-xL (an important protein involved in the cross talk of apoptosis and autophagy)^23^. Furthermore, BP-1-102 has been shown to have significant effects in several disease models^22–24^. However, the effect of BP-1-102 on vascular inflammation and AAA progression has not been reported. Based on the evidences mentioned above, we hypothesized that BP-1-102 could attenuate AAA progression by suppressing the JAK2/STAT3 and NF-κB signaling pathways and maintaining autophagy.

## Materials and methods

### Animals

ApoE^−/−^ mice (4–6 weeks old, male) were purchased from BEIJING HUAFUKANG Bioscience CO. INC. All mice were cared for in accordance with the Guide of the Care and Use of Laboratory Animals published by the U.S. National Institutes of Health (Publication No. 85-23). All procedures and experiments were approved by the Animal Experiment Advisory Committee of the Second Xiang-ya hospital of Central South University.

### AAA establishment

8-week-old ApoE^−/−^ mice were anaesthetized (Toletil 20 (20 mg/kg) & xylazine (5 mg/kg), i.p.). All ApoE^−/−^ mice were randomly divided into three groups: the mock group (mice with only saline pump infusion), AngII^+^ group (mice with AngII pump infusion and DSMO-PBS administration), AngII^+^ BP-1-102^+^ group (mice with AngII infusion and BP-1-102 administration). For AAA induction, mice were infused with AngII (1.44 mg/kg/day) (Sigma-Aldrich, St Louis, MO) that was subcutaneously delivered by an Alzet model 2004 osmotic minipump (DURECT Corp., Cupertino, CA, USA)^25^. The STAT3 inhibitor BP-1-102 (Selleck, 5 mg/kg) (dissolved in 0.1% DMSO in PBS) was administrated orally every other day to ApoE^−/−^ mice with AngII infusion. Mice were sacrificed on day 7, day 14, and day 28, respectively, to observe the effects of BP-1-102 on AAA formation and progression (*n* = 8 per group).The length of aorta from thoracic to abdominal cavity was exposed and photographed carefully using an LEICA DFC450C attached to an LEICA M165FC with the Leica Application Suite software (LAS 4.5.0). Digital image analysis software (ImageJ v.1.41, National Institutes of Health) was used to measure the maximum aortic diameter in the infra-diaphragm by calibrating with ruler correction. Aorta tissues with maximum diameter were harvested for further investigations. A commonly used clinical standard to diagnose AAA is an increase in aortic diameter of ≈50%^26^. The average diameter of normal suprarenal aorta in native control mice is ≈0.8 ± 0.01 mm. We therefore set a threshold of 1.25 mm as evidence of the incidence of aneurysm formation.

### VSMCs culture

Murine vascular smooth muscle cells (VSMCs) were acquired from the aorta of ApoE^−/−^ mice by a previously established method^27^. VSMCs were cultured in Dulbecco’s modified Eagle’s medium containing 10% fetal bovine serum at 37 °C under a humidified atmosphere containing 5% CO_2_. Cells passaged within six times were used in the following experiments. 1 × 10^5^/well VSMCs were seeded to 6-well plate 1 day before adding reagents. VSMCs were treated with AngII (10 μM) for 24 h or 48 h; or with BP-1-102 (0.05 μM), BP-1-102 (0.1 μM), BP-1-102 (0.5 μM) for 24 h; or with AngII (10 μM) + BP-1-102 (0.05 μM), AngII (10 μM) + BP-1-102 (0.1 μM), AngII (10 μM) + BP-1-102 (0.5 μM) for 24 h, respectively. BP-1-102 was dissolved in 99% DMSO to make stock solution. Untreated VSMCs were used as control group. VSMCs in each group were collected for further analysis.

### Hematoxylin & eosin staining

The part of the aorta with a maximum diameter was harvested. Aorta tissues were embedded in Tissue-Tek II OCT compound (SAKURA 4583, SAKURA FINETEK USA INC). 5 mm sections were made and stained with hematoxylin & eosin (HE). Samples were photographed using Axio Scope. A1 (FL) (Carl Zeiss).

### Elastin volume measurement

The elastin amounts of the part of aortas with maximum diameter harvested from ApoE^−/−^ mice were quantified using a Fastin elastin assay kit (Biocolor, County Antrim, UK) according to the manufacturer’s instructions. Briefly, the aortic tissues were weighed and cut into pieces with scissors. To convert insoluble elastin to water soluble alpha-elastin, ~1 mg (dry weight) of samples were placed with 300 μL of 0.25 M oxalic acid into a metal heating block with the thermostat set at 100 °C for 60 min. The samples were then centrifuged at 3000 × *g* for 10 mins and 250 μL of the supernatant was retained in a new EP tube with 250 μL of elastin precipitating reagent. The tubes were centrifuged at 10,000 × *g* for 10 mins and the supernatants were discarded, followed by adding 1.0 mL of the dye reagent, and were placed in a mechanical shaker at room temperature for 90 mins. Subsequently, the tubes were centrifuged at 10,000 × *g* for 10 mins and the supernatants discarded. 250 μL of dye dissociation reagent was added to the elastin-bound dye pellet to release the bound dye into solution. Each sample (250 μL) were transferred to the wells of 96-well plate and the optical density was measured at 513 nm. Elastin values were standardized to the corresponding dry weight.

### RNA extraction, cDNA synthesis, and qRT-PCR

Total RNA was extracted from homogenized macrophages cells using the TaKaRa MiniBEST Universal RNA Extraction Kit (Takara, 9767) according to the manufacturer’s instructions. cDNA was synthesized using PrimeScript™ RT reagent Kit with gDNA Eraser (Perfect Real Time) (Takara, RR047A). Real-time PCR was performed using TB Green™ Premix Ex Taq™ II (Tli RNaseH Plus) (Takara RR820A) and Light Cycler/LightCycler480 System (Roche Diagnostics) according to the manufacturer’s instructions. Expression levels of the target genes were normalized to that of β-actin. Primers sequences used were listed as follows:

MMP-2: Forward (5′−3′) CCAGATGTGGCCAACTACAA; Reverse (5′−3′) GGCATCATCCACTGTCTCTG;

MMP-9: Forward (5′−3′) GCAGAGATGCGTGGAGAGT; Reverse (5′−3′) ATGTTGTGGTGGTGCCACTTC; β-Actin is Forward (5′−3′) CACGAAACTACCTTCAACTC; Reverse (5′–3′) CATACTCCTGCTTGCTGATCC.

### Transmission electron microscopy

Aorta tissue samples were sliced into 1 mm^3^ sections, prefixed via immersion in chilled 2.5% glutaraldehyde in phosphate buffer (pH 7.2) on ice for 1 h, fixed in osmium tetraoxide (Servivebio, G1102) for 1 h and then dehydrated for 10 min each in a series of 50, 70, 80, 90, and 100% ethanol. Next, the samples were dehydrated three times with propylene oxide for 10 min each, infiltrated for 10 min with propylene oxide andepoxy resin (vol/vol = 1:1), embedded with EPON 812 epoxy resin, DDSA, DMP30 and MNA resin, and aggregated for 24–48 h at 60 °C. After polymerization, 70 nm ultrathin sections were prepared using a diamond knife and Reichert Nissei Ultracuts (Leica). Sections were then stained with uranyl acetate and lead staining solution (Sigma-Aldrich). The stained sections were photographed with a transmission electron microscope (HITACHI, HT7700, Japan).

### Western blotting

The western blotting analysis was performed as previously described^28^. Briefly, cells/aortic tissue were harvested and homogenized on ice with RIPA (Beyotime Biotechnology P0013B) containing proteinase inhibitor (Complete, EDTA-free protease inhibitor cocktail Tablets provided in EASY pack, Roche, 04 693 132 001). An equivalent amount of the cell lysate was subjected to sodium dodecyl sulfate polyacrylamide gel electrophoresis (SDS-PAGE) and then transferred for the western blotting assay. The polyvinylidene fluoride (PVDF) membranes were incubated with Jak2 (D2E12) XP® Rabbit mAb (#3230, Cell Signaling Technology), Stat3 (124H6) Mouse mAb (#9139, Cell Signaling Technology), NF-κB p65 (D14E12) XP® Rabbit mAb (#8242, Cell Signaling Technology), Phospho-NF-κB p65 (Ser536) (93H1) Rabbit mAb (#3033, Cell Signaling Technology), LC3A/B (D3U4C) XP® Rabbit mAb (#12741, Cell Signaling Technology), Bcl-xL (54H6) Rabbit mAb (#2764, Cell Signaling Technology), Bcl-2 (50E3) Rabbit mAb (#2870, Cell Signaling Technology), Beclin-1 Antibody (#3738, Cell Signaling Technology), β-Actin (8H10D10) Mouse mAb (#3700, Cell Signaling Technology) primary antibodies and the appropriate horseradish peroxidase (HRP)-linked anti-rabbit/mouse IgG secondary antibodies (Cell Signaling Technology) according to the primary antibody species. The membranes were treated with an enhanced chemiluminescent detection kit (Thermo Fisher Scientific, #17097) and the signals were collected and photographed (Bio-Rad, Gel Doc, XR). All experiments were repeated 3 times. The bands were quantified by densitometry with the Scion Image software (ImageJ 1.42q; NIH, Bethesda, MD).

### Immunostaining

LC3 expressions were analyzed with immunostaining in aortas. The part of aorta with maximum diameter aortas (*n* = 8 per group) were harvested collected and embedded in Tissu-Tek O.C.T. compound (SAKURA 4583, SAKURA FINETEK USA INC.) The samples were frozen at −80 °C. Then, 5-μm sections were fixed in pre-cold 4% formaldehyde for 15–20 min. After fixation, samples were blocked in 5% BSA in PBS for 1 h. After blocking, samples were incubated with LC3B (E5Q2K) mouse mAb (#83506, Cell Signaling Technology, USA) and rabbit anti-alpha smooth muscle actin antibody (#ab124964, Abcam, Cambridge, USA) at 4 °C for 12 h. The secondary antibodies including Alexa, Fluor 488 conjugated anti-mouse IgG (H + L) (#4408, Cell Signaling Technology, Boston, MA, USA) and Alexa, Fluor 647 conjugated anti-rabbit IgG (H + L) (#4414, Cell Signaling Technology, Boston, MA, USA) were used for detection. Nuclei was stained with 40, 6-diamidino-2-phenyindole, DAPI (Vector Laboratories, Burlingame, CA, USA). ProLong™ Gold Antifade Mountant (Thermo Fisher, P36930) was applied to protect the fluorescence from fading. Images were taken using a Carl Zeiss Axio Imager AX10 with ZEN 2011 (blue edition). The fluorescence was measured using digital image analysis software (ImageJ v.1.41, National Institutes of Health).

### Cytokines measurement

Aortas were snap-frozen and analyzed using a Proteome Profiler Mouse Cytokine Array Kit (R&D Systems, Minneapolis, MN, USA). The tissues were processed according to the manufacturer’s protocol. The densitometric volume was determined by spectrophotometry using Thermo Scientific software (Thermo Fisher Scientific, Waltham, MA, USA). All the procedures were performed strictly according to the manufacturers’ instructions.

### Tunel staining

The apoptosis of cells in AAA tissue was analyzed with In situ Cell Death Detection Kit, POD (Roche, 11684817910). All the procedures were performed strictly according to the manufacturers’ instructions.

### Flow cytometry

Annexin-V-PE (fluorescein isothiocyanate)/propidium iodide (PI) staining was performed using Annexin-V-PE/PI apoptosis detection kit (BD 559763) according to the manufacturer’s instruction. VSMCs were harvested then suspended in binding buffer. 1.0 × 10^5^ cells in 100 µL binding buffer were added into a 5 mL tube. Also, 5 µL Annexin-V-PE reagent and 5 µL PI were added into each tube. The mixture was incubated in the dark at room temperature for 15 mins. Then 400 µL phosphate buffered saline (PBS) was added into each tube. The samples were analyzed by flow cytometry (FACS; FACS Calibur, BD Biosciences, San Jose, California, USA). Flowjo7.6.1 software was used for quantification of cell apoptosis. All experiments were performed in triplicate and repeated at least three times.

### Statistics analysis

SPSS 22.0 for Windows (SPSS, Chicago, IL, USA) was used for all statistics. Data are expressed as mean ± standard deviation (SD). Statistical significance of differences between two groups was calculated by chi‐square test or unpaired *t* test, as appropriate. Comparisons were made using a one-way or two-way ANOVA with the Tukey’s HSD post hoc analysis for multiple comparisons. Values were considered significantly different when *p* < 0.01.

## Results

### BP-1-102 decreased AAA formation and progression

**Fig. 1:**
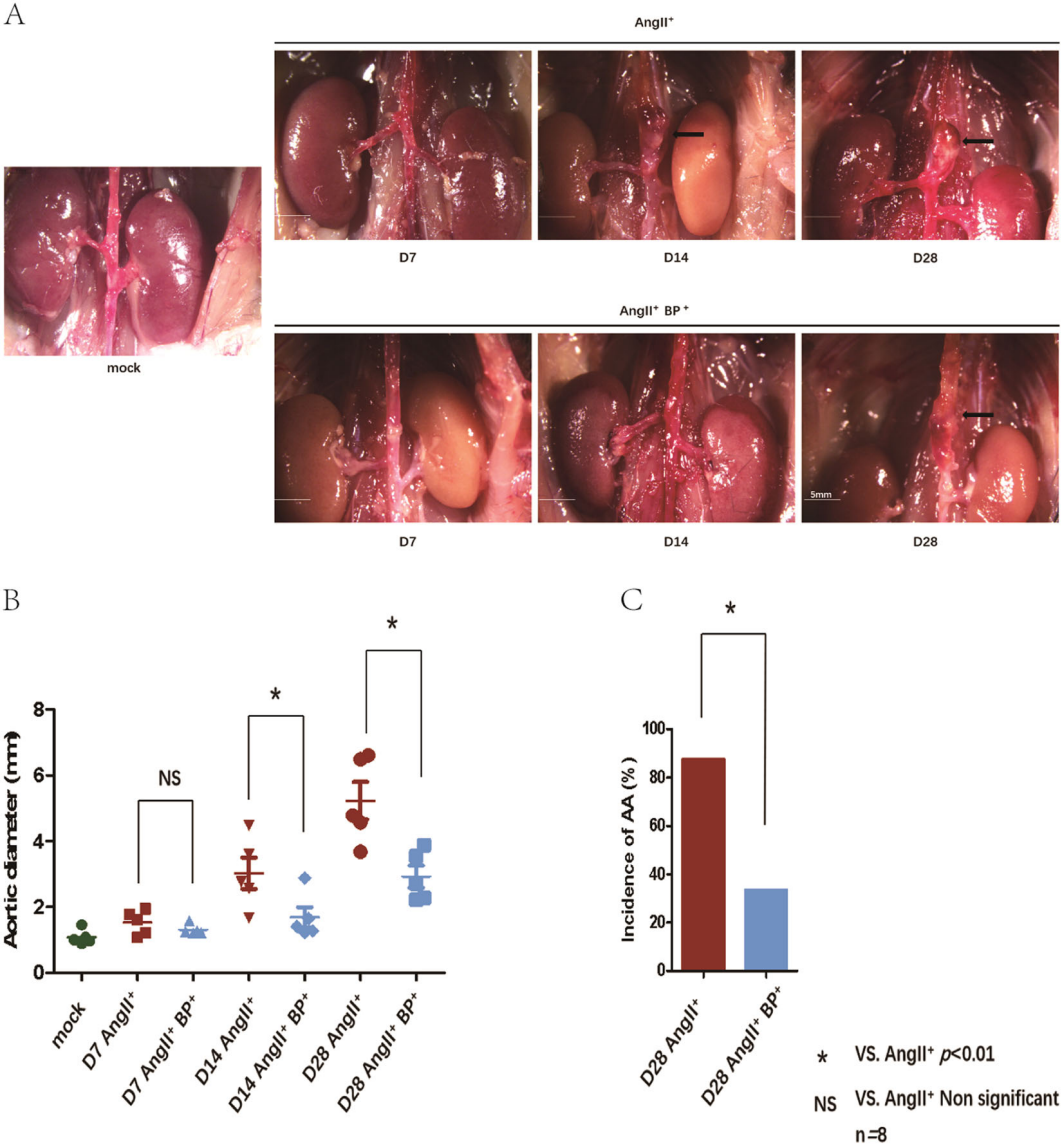
BP-1-102 decreased AAA formation and progression. **a** Eight-week-old male ApoE^−/−^ mice were administrated with AngII (1.44 mg/kg/day) and DSMO-PBS (AngII^+^ group) or AngII (1.44 mg/kg/day) and BP-1-102 (5 mg/kg) (AngII^+^ BP-1-102^+^ group) at indicated time points. Saline administrated was used as control group (mock) (left). Representative images of AAA at each time point were presented. AngII^+^ BP-1-102^+^ group (AngII^+^BP^+^, lower panel) showed less severity of AAA compared with AngII^+^ group (upper panel) at each time point. Arrows indicate typical AAA. **b** The maximum aortic diameter at the infra-diaphragm was measured. The AngII^+^ BP-1-102^+^ group (AngII^+^BP^+^, blue dots) showed reduced aortic diameter comparing with AngII^+^ (red dots) at each time point (day 7, day 14, and day 28). The results were presented as mean ± standard deviation (SD). *n* *=* 8. *P < 0.01 vs. AngII^+^ group (two-way ANOVA followed by Tukey’s test). *NS* indicates non-significant differences compared with AngII^+^ group. **c** The incidence of AAA was evaluated on day 28. The AngII^+^ BP-1-102^+^ group (blue column) showed reduced incidence of AAA compared with AngII^+^ group (red column). *n* *=* 8. *P < 0.01 vs. AngII^+^ group (chi‐square test). *NS* indicates non-significant differences compared with AngII^+^ group.

To test whether STAT3 inhibitor BP-1-102 alleviates AAA growth, mice were sacrificed on day 7, day 14, and day 28, respectively. Representative images of the aorta at each time point from the different group were presented (Fig. 1a). The mean of the maximum aortic diameter on day 14 and day 28 in AngII^+^ BP-1-102^+^ group were significantly smaller (1.688 ± 0.67 mm, 2.928 ± 0.75 mm) than that in the corresponding AngII^+^ group (3.026 ± 1.07 mm, 5.226 ± 1.28 mm) (Fig. 1b). The incidence of AAA on day 28 was markedly decreased in AngII^+^ BP-1-102^+^ group (33.33%) comparing with AngII^+^ group (87.5%) (Fig. 1c).

### BP-1-102 maintained aortic elastin structure and volume, and reduced MMPs expression

**Fig. 2:**
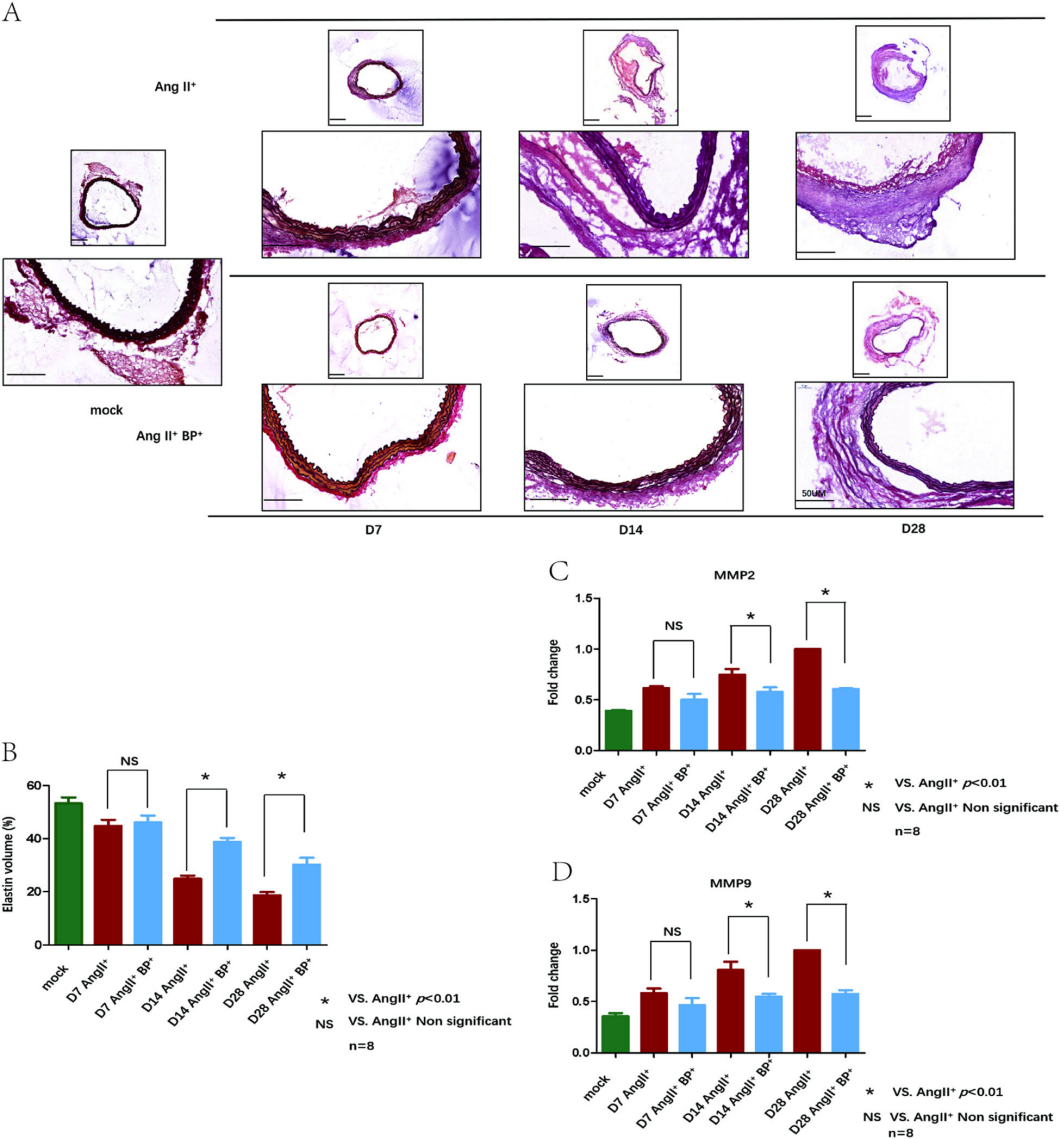
BP-1-102 maintained elastin structure and volume, and reduced MMPs expressions. **a**. Elastin structure of aortic wall was evaluated at indicated time points using Weigert’s resorcin-fuchsin elastic staining. Saline treated group was used as control (mock) (left). Elastin degradation was prevented in AngII^+^ BP-1-102^+^ group (AngII^+^BP^+^, lower panel) compared with AngII^+^ group (upper panel) at each time point. **b**. Mean aortic wall elastin volume of AngII^+^ group (red column) and AngII^+^ BP-1-102^+^ group (AngII^+^BP^+^, blue column) were analyzed at indicated time points. Saline treated group was used as control (green column). Comparing with AngII^+^ group, the AngII^+^ BP-1-102^+^ group showed preserved elastin volume at each time point. The results were presented as mean ± standard deviation (SD). *n* *=* 8. *P < 0.01 vs. AngII^+^ group (two-way ANOVA followed by Tukey’s test). *NS* indicates non-significant differences compared with AngII^+^ group. **c** mRNA expression of MMP-2 was determined using real-time PCR. Fold changes were calculated and statistically analyzed at indicated time points. Saline treated group were used as control (green column). The results were presented as mean ± standard deviation (SD). *n* *=* 8. *P < 0.01 vs. AngII^+^ group (two-way ANOVA followed by Tukey’s test). *NS* indicates non-significant differences compared with AngII^+^ group. **d** mRNA expression of MMP-9 was determined using real-time PCR. Fold changes were calculated and statistically analyzed at indicated time points. Saline treated group were used as control (green column). The results were presented as mean ± standard deviation (SD). *n* *=* 8. *P < 0.01 vs. AngII^+^ group (two-way ANOVA followed by Tukey’s test). *NS* indicates non-significant differences compared with AngII^+^ group.

Representative images of elastic lamellae using Weigert’s resorcin-fuchsin elastic staining were shown in Fig. 2a. Considerable destructions of elastic lamellae were detected in AngII^+^ group, while much less loss of the elastic lamellae was observed in AngII^+^ BP-1-102^+^ group at corresponding time points. Elastin volume was further determined. Comparing with AngII^+^ group (day 7, day 14, and day 28: 44.79% ± 6.66, 24.86% ± 3.45, and 18.74% ± 3.19), AngII^+^ BP-1-102^+^ group (day 7, day 14, and day 28: 46.16% ± 7.19, 38.74% ± 4.41, and 30.24% ± 7.36) showed preserved elastin volume on day 14 and day 28 (Fig. 2b). MMPs contribute to the initiation and progression of AAA. mRNA expressions of MMP-2 and MMP-9 were determined with real-time PCR. AngII significantly increased expressions of MMP-2 and MMP-9 in ApoE^−/−^ mice while treatment with BP-1-102 reversed these effects at indicated time points (Fig. 2c, d).

### BP-1-102 inhibited inflammatory cells infiltration and suppressed the expressions of pro-inflammatory cytokines

**Fig. 3:**
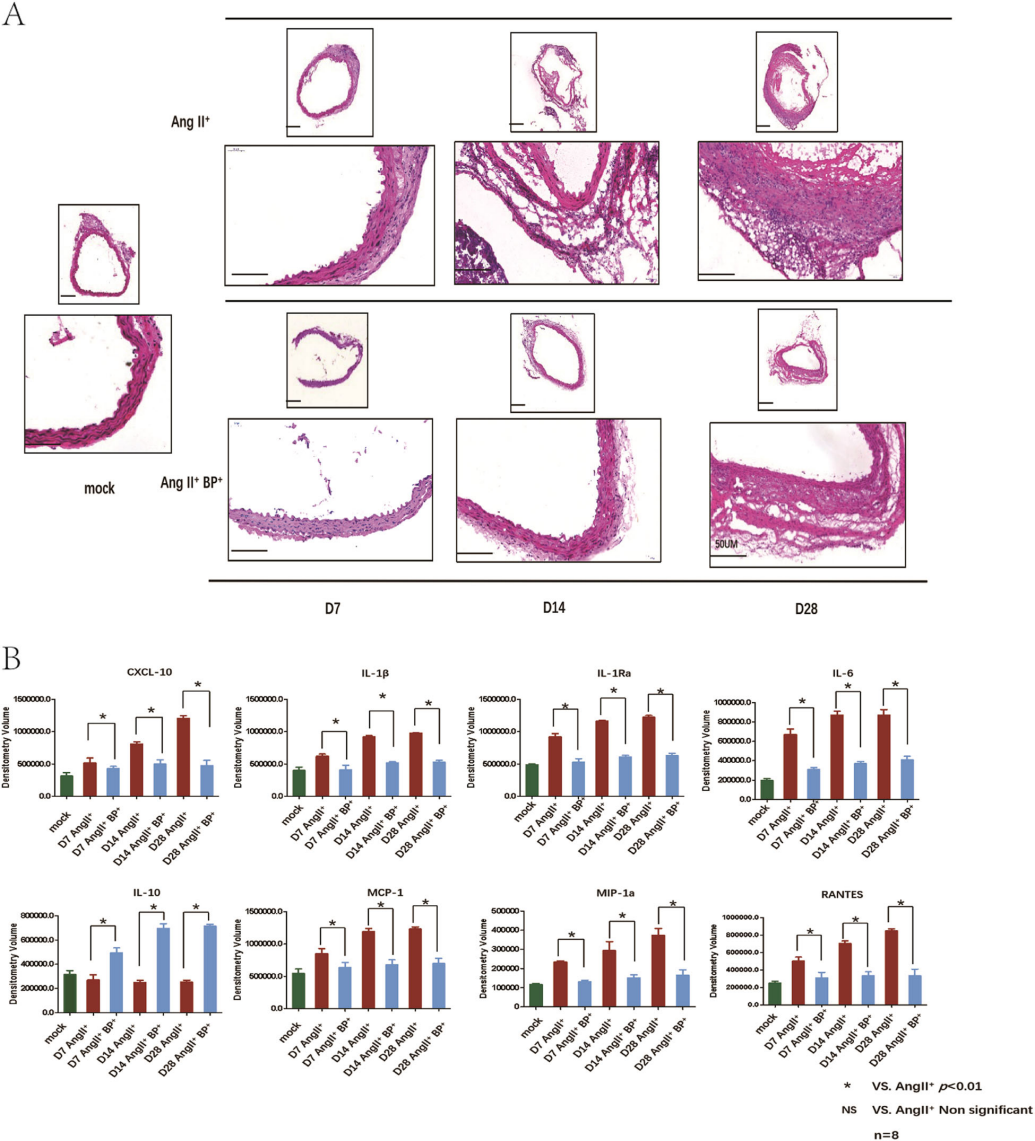
BP-1-102 inhibited inflammatory cells infiltration and suppressed pro-inflammatory cytokines expression. **a** The part of aorta with maximum diameter aorta was collected and embedded. Five-micrometer sections were made for HE staining. Representative histology images of AAA at indicated time points were shown. The integrity of aortic wall in AngII^+^ BP-1-102^+^ group (AngII^+^BP^+^), were maintained comparing with that of AngII^+^ group, while the inflammatory cells infiltrations were reduced in AngII^+^ BP-1-102^+^ group comparing with that of AngII^+^ group. **b** Inflammation-related cytokines expression in AAA tissues were determined using proteome profiler Mouse cytokine Array kit. Densitometry volume of each cytokine was determined and statistically analyzed. CXCL-10, IL-1β, IL-1Rα, IL-6, MCP-1, MIP-1α, and RANTES were all decreased in AngII^+^ BP-1-102^+^ group (AngII^+^BP^+^, blue column) compared with AngII^+^ group (red column) at each time point. While the anti-inflammation factor IL-10 was significantly increased in AngII^+^ BP-1-102^+^ group (AngII^+^BP^+^), compared with the AngII^+^ group. Saline treated group were used as control (green column). The results were presented as mean ± standard deviation (SD). *n* *=* 8. *P < 0.01 vs. AngII^+^ group (two-way ANOVA followed by Tukey’s test). *NS* indicates non-significant differences compared with AngII^+^ group.

The histopathology of experimental AAA in ApoE^−/−^ mice was evaluated by HE staining. Inflammatory cells including lymphocytes, monocytes, neutrophils, and macrophages, are important players in vascular inflammation and AAA formation. AngII resulted in destructive structure of aortic wall and numerous inflammatory cells infiltration in aortic wall whereas these changes were relatively mild with the treatment of BP-1-102 (Fig. 3a). Cytokines expressions in the AAA tissues at each time point were determined using Proteome Profiler Mouse Cytokine Array Kit. Cytokines involved in immune-regulatory, chemotactic activities, and inflammatory responses including CXCL-10, IL-1β, IL-1Rα, RANTES (CCL5), IL-6, MCP-1(CCL2), and MIP-1α were all significantly decreased in the AAA tissues from AngII^+^BP-1-102^+^ group compared with AngII^+^ group, while the anti-inflammation factor (IL-10) was increased in AngII^+^ BP-1-102^+^ group compared with the AngII^+^ group (Fig. 3b).

### BP-1-102 reduced the expression of STAT3 and NF-κB signaling pathways-related proteins in AAA tissues

**Fig. 4:**
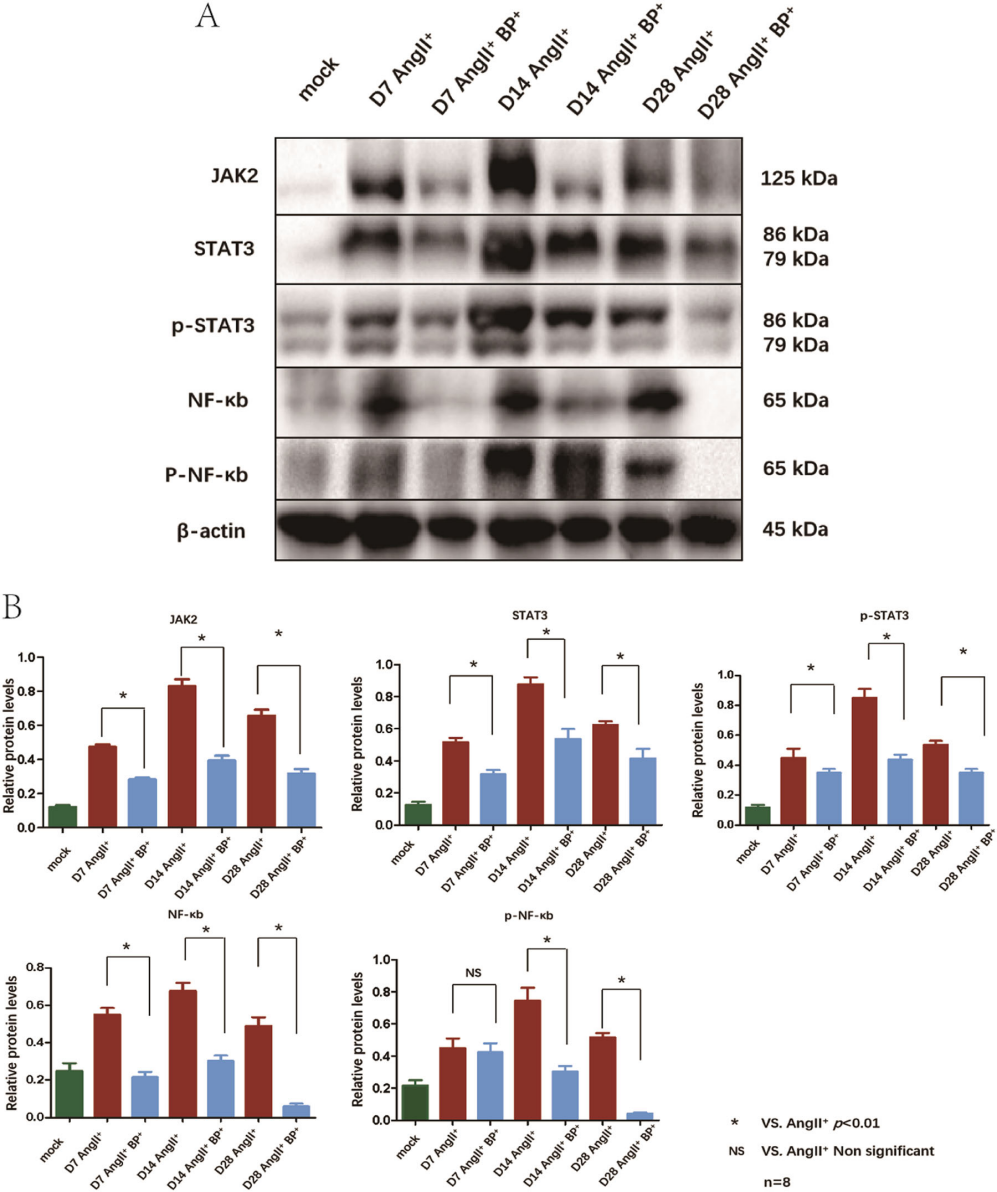
BP-1-102 reduced the expression of inflammation signaling pathways-related proteins in AAA tissues. **a** Western blotting was applied to analyze inflammation signaling pathways-related proteins expressions in AAA tissue at indicated time points. JAK2, STAT3, p-STAT3, NF-κB, and p-NF-κB expressions were evaluated in AngII^+^ group and compared with AngII^+^ BP-1-102^+^ group (AngII^+^BP^+^). AngII^+^ BP-1-102^+^ group showed reduced inflammation signaling pathways-related proteins compared with AngII^+^ group at each time point. Saline treated group was used as control (mock). **b** Relative protein levels of JAK2, STAT3, p-STAT3, NF-κB, p-NF-κB were determined by normalized with β-actin expression in AngII^+^ group and AngII^+^ BP-1-102^+^ group (AngII^+^BP^+^) at indicated time points. Saline treated group was used as control (green column). AngII^+^ BP-1-102^+^ group (blue column) showed reduced inflammation signaling pathways-related proteins compared with AngII^+^ group (red column) at each time point. The results were presented as mean ± standard deviation (SD). *n* *=* 8. *P < 0.01 vs. AngII^+^ group (two-way ANOVA followed by Tukey’s test). *NS* indicates non-significant differences compared with AngII^+^ group.

As mentioned in the part of introduction, we focused on NF-κB and STAT3 signaling pathways in the present study. The two pathways-related proteins expressions (JAK2, STAT3, p-STAT3, NF-κB, and p-NF-κB) in AAA tissues at each time point were evaluated using western blotting. AngII resulted in elevated expression of JAK2, STAT3, p-STAT3, NF-κB, and p-NF-κB. BP-1-102 administration reduced the expression levels of JAK2, STAT3 (on day 7, day 14, and day 28), and p-STAT3 (on day 14 and day 28) in AngII^+^ BP-1-102^+^ group comparing with AngII^+^ group. Similarly, BP-1-102 treatment decreased the expression levels of NF-κB (on day 7, day 14, and day 28), and p-NF-κB (on day 14 and day 28) in AngII^+^ BP-1-102^+^ group comparing with AngII^+^ group (Fig. 4a, b).

### BP-1-102 regulated autophagy and apoptosis in AAA tissues

**Fig. 5:**
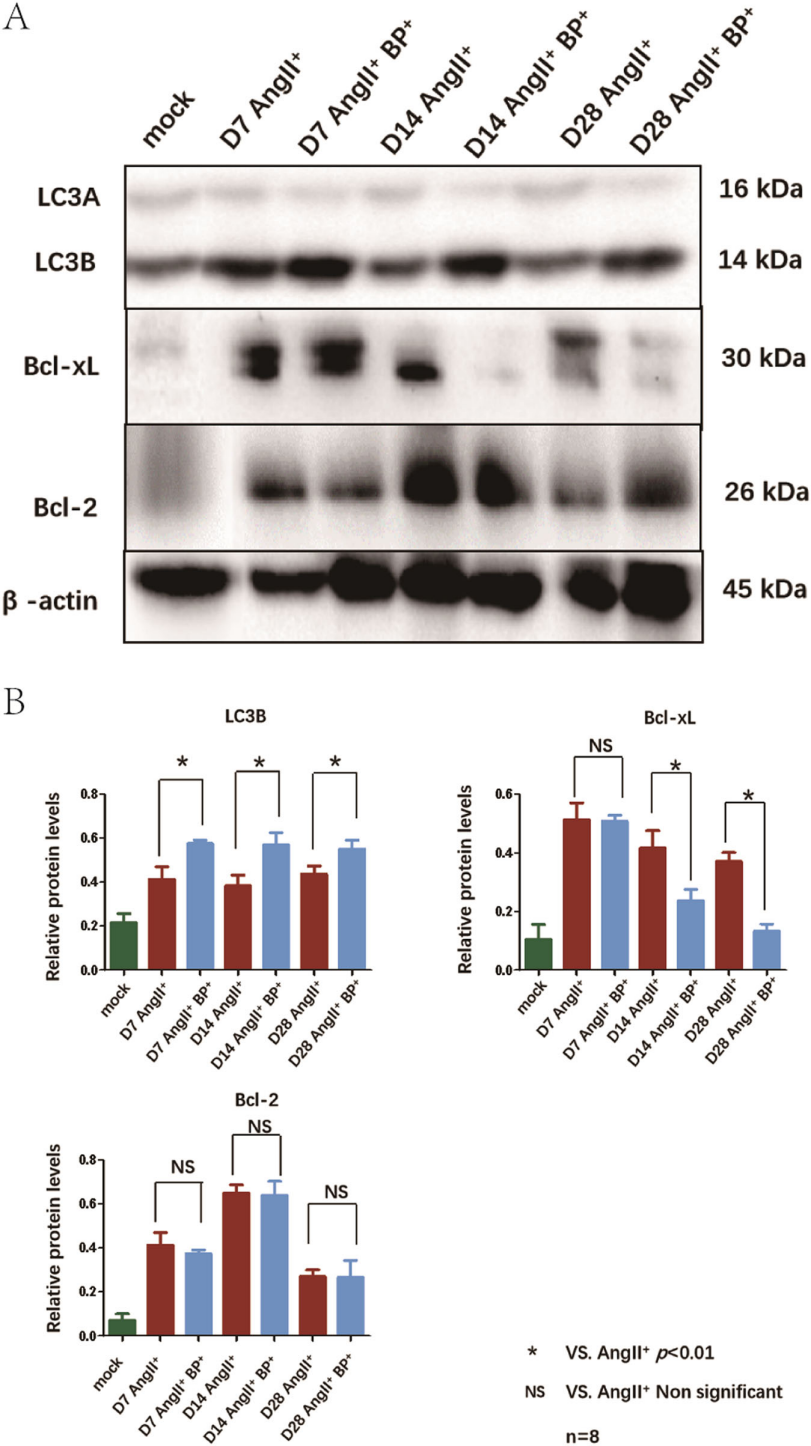
BP-1-102 regulated autophagy-related proteins expression in AAA tissues. **a** Autophagy and apoptosis related proteins expression in AAA tissue were evaluated with western blotting. LC3, Bcl-xL, and Bcl-2 expressions were evaluated in AngII^+^ BP-1-102^+^ group (AngII^+^BP^+^) and compared with AngII^+^ group at indicated time points. **b** Relative protein levels of LC3B, Bcl-xL, and Bcl-2 were determined by normalized with β-actin expression at indicated time points. Levels of LC3B, Bcl-xL, and Bcl-2 in AngII^+^ BP-1-102^+^ group (AngII^+^BP^+^, blue column) were compared with that of AngII^+^ group (red column). Saline treated group was used as control (green column). The results were presented as mean ± standard deviation (SD). *n* *=* 8. *P < 0.01 vs. AngII^+^ group (two-way ANOVA followed by Tukey’s test). *NS* indicates non-significant differences compared with AngII^+^ group.

**Fig. 6:**
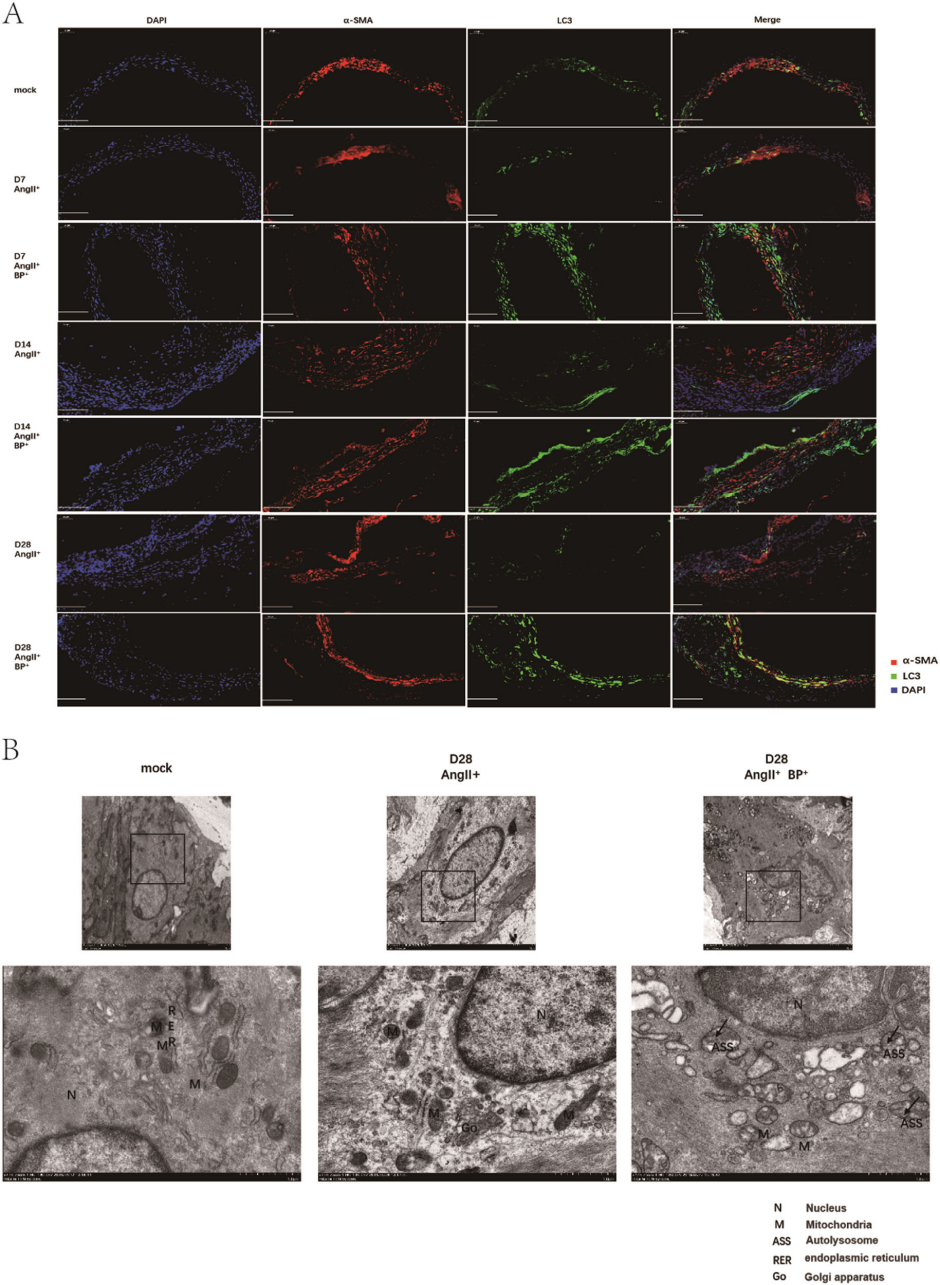
BP-1-102 maintained LC3B expression and autolysosome in AAA tissues. **a** LC3B (green) expression in AAA tissues was investigated using immunofluorescence staining at indicated time points. α-SMA (red) was used to stain VSMCs. DAPI (blue) was used to stain cell nuclei. Saline treated group was used as control (mock). **b** TEM was used to investigate the ultrastructure of mitochondria (M), ASS (atuolysosome), AP (autophagosome) changes in VSMCs of aortic wall on day 28. AngII^+^ BP-1-102^+^ group (AngII^+^BP^+^, right panel) showed increased ASS comparing with AngII^+^ group (middle panel). N (nucleus), RER (endoplasmic reticulum), Go (Golgi apparatus). Saline treated group was used as control (mock) (left panel).

Autophagy and apoptosis related proteins expression in AAA tissues were determined using western blotting. LC3B was upregulated in AngII^+^ BP-1-102^+^ group comparing with AngII^+^ group. We did not observe significant differences of Bcl-2 expression between AngII^+^ BP-1-102^+^ group and AngII^+^ group. Bcl-xL expression was significantly suppressed in AngII^+^ BP-1-102^+^ group comparing with AngII^+^ group (Fig. 5a, b). The indicator of autophagy, LC3 expression was further investigated using immunofluorence. We observed that LC3 expression in VSMCs was maintained in AngII^+^ BP-1-102^+^ group comparing with AngII^+^ group at each time point (Fig. 6a). TEM was applied to observe the ultrastructure of VSMCs. We observed autolysosome was reduced on day 28 which indicated that autophagy was reduced at later stage of AAA formation. In the AngII^+^ BP-1-102^+^ group, autolysosome was observed on day 28 which suggested that autophagy was maintained by BP-1-102 (Fig. 6b). The apoptosis of cells in AAA tissue was further analyzed with in situ Cell Death Detection Kit. We found that AngII could induce apoptosis in AAA tissue during AAA formation, especially at later stage (day 14 and day 28). BP-1-102 administration could reduce VSMCs apoptosis (Supplementary Fig. 1).

### BP-1-102 inhibited AngII-induced JAK2/STAT3 and NF-κB signaling activation in VSMCs

**Fig. 7:**
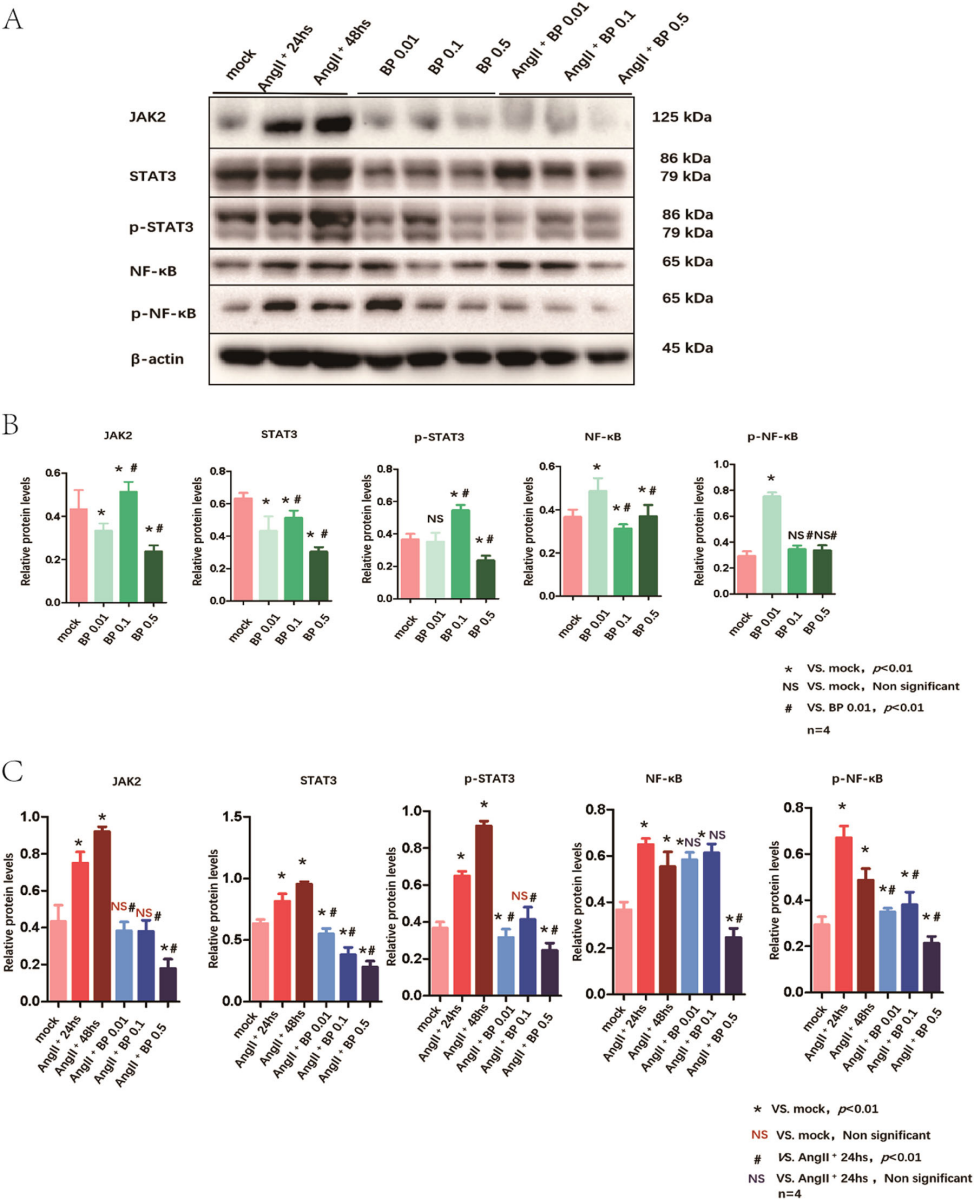
BP-1-102 inhibited AngII-induced JAK2/STAT3 and NF-kB signaling activation in VSMCs. **a** VSMCs were treated with AngII (10 μM) for 24 and 48 h. JAK2/STAT3 and NF-kB signaling pathway related proteins expression were evaluated and compared with untreated VSMCs. VSMCs were treated with BP-1-102 (0.01–0.5 μM) for 24 h. JAK2/STAT3 and NF-kB signaling pathway related proteins expression were evaluated. VSMCs were treated with AngII (10 μM) + BP-1-102 (0.01–0.5 μM) for 24 h. **b** Relative protein levels of were determined by normalized with β-actin expression in BP-1-102 (0.01–0.5 μM) groups, respectively, and compared with untreated VSMCs (mock) or with BP-1-102 (0.01 μM) group. The results were presented as mean ± standard deviation (SD). *n* *=* 4, *P < 0.01 compared with Mock. *NS* indicates non-significant differences compared with Mock. ^**#**^*P* < 0.01 compared with BP-1-102 (0.01 μM) group (two-way ANOVA followed by Tukey’s test). **c** Relative protein levels were determined by normalized with β-actin expression in AngII (10 μM) (24 or 48 h) groups, AngII (10 μM) + BP-1-102 (0.01–0.5 μM) groups, respectively, and compared with untreated VSMCs (mock) or with AngII (10 μM) for 24 h group. The results were presented as mean ± standard deviation (SD). *n* *=* 4, *P < 0.01 compared with Mock. ^**#**^*P* < 0.01 compared with AngII (10 μM) 24 h group. *NS* indicates non-significant differences compared with AngII (10 μM) 24 h group (two-way ANOVA followed by Tukey’s test).

VSMCs are major component of the aortic wall and play a central role on vascular inflammation and AAA formation. We used VSMCs to study the role of BP-1-102 in the pathogenesis of AAA. VSMCs were first treated with AngII (10 μM) for 24 and 48 h. Comparing with mock group, pathways-related proteins: JAK2, STAT3, p-STAT3, NF-κB, and p-NF-κB were all upregulated after 24 and 48 h induction. Using different concentration of BP-1-102 (0.01, 0.1, and 0.5 μM) to treat VSMCs for 24 h, we found that it could suppress the expression of JAK2/STAT3 and NF-κB, and these effects were concentration-dependent (Fig. 7a, b). We further observed the effects of different concentrations of BP-1-102 (0.01, 0.1, and 0.5 μM) on VSMCs with AngII. Our data showed that it also blocked the AngII-induced JA2K/STAT3 and NF-κB signaling activation in a concentration-dependent manner (Fig. 7a, c).

### BP-1-102 maintained the autophagy-related proteins expression in VSMCs

**Fig. 8:**
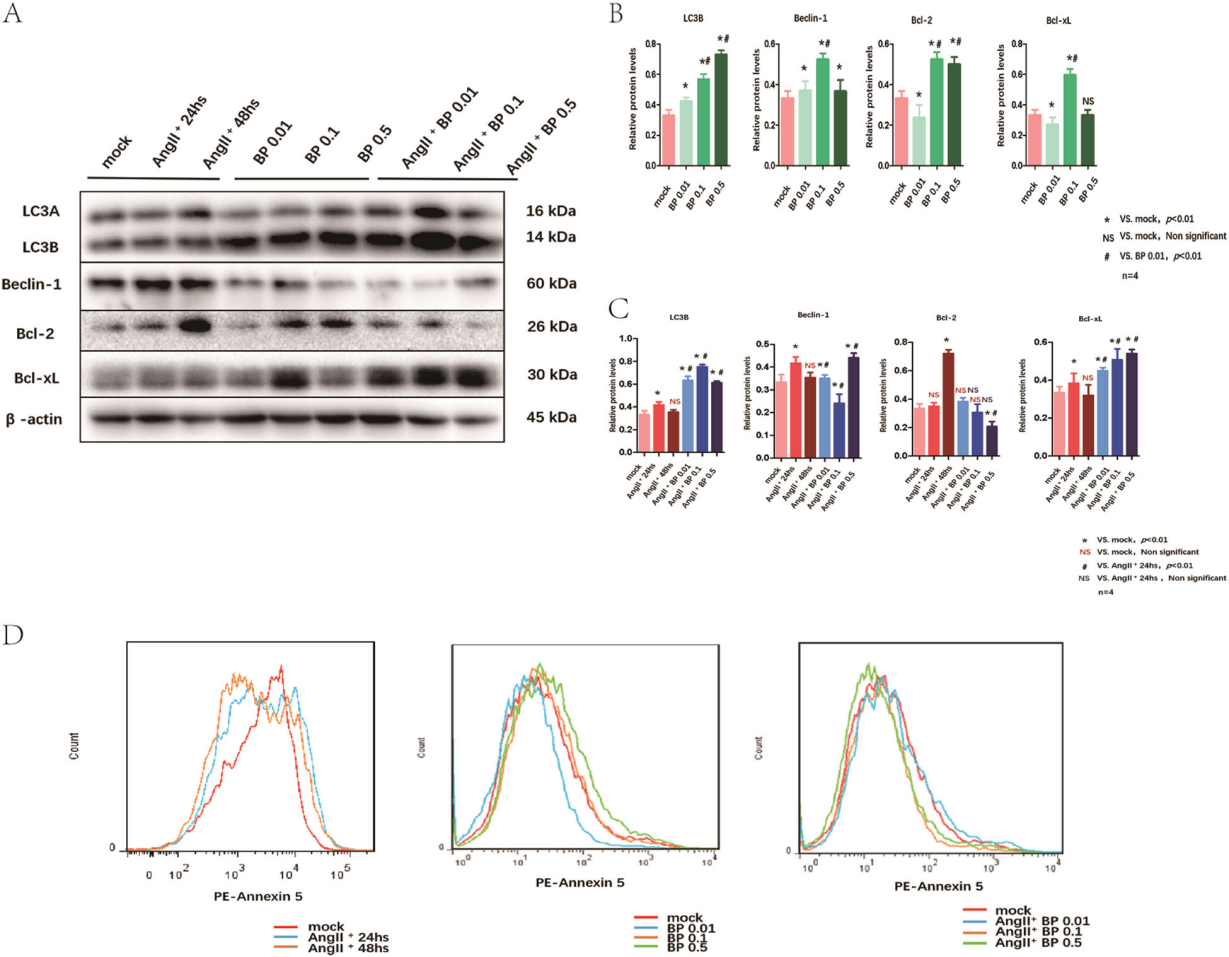
BP-1-102 maintained the autophagy-related proteins expression in VSMCs. **a** Autophagy-related proteins expression in VSMCs treated with AngII (10 μM) for 24 or 48 h were evaluated, compared with untreated (mock group) (left panel). VSMCs were treated with BP-1-102 (0.01–0.5 μM) for 24 h (middle panel). VSMCs were treated with AngII (10 μM) + BP-1-102 (0.01 μM-0.5 μM) for 24 h (right panel). Autophagy-related proteins expression were evaluated using western blotting. **b** Relative protein levels were determined by normalized with β-actin expression in BP-1-102 (0.01–0.5 μM) groups, respectively, and compared with untreated VSMCs (mock) or with BP-1-102 (0.01 μM) group. *n* *=* 4, *P < 0.01 compared with Mock. *NS* non-significant differences compared with Mock. ^**#**^*P* < 0.01 compared with BP-1-102 (0.01 μM) group (two-way ANOVA followed by Tukey’s test). **c** Relative protein levels were determined by normalized with β-actin expression in AngII (10 μM) (24 or 48 h) groups, AngII (10 μM) + BP-1-102 (0.01–0.5 μM) groups, respectively, and compared with untreated VSMCs (mock) or with AngII (10 μM) (24 h). *n* *=* 4, *P < 0.01 compared with Mock. *NS (red)* non-significant differences compared with Mock. ^**#**^*P* < 0.01 compared with AngII (10 μM) for 24 h group. *NS (blue)* non-significant differences compared with AngII (10 μM) for 24 h group (two-way ANOVA followed by Tukey’s test). **d** Annexin-V-PE/PI staining was performed using flow cytometry to investigate apoptosis of VSMCs treated with AngII (10 μM) for 24 or 48 h and compared with untreated VSMCs (mock, upper left). VSMCs were treated with BP-1-102 (0.01–0.5 μM) and compared with untreated VSMCs (mock, lower left). VSMCs were treated with AngII (10 μM) + BP-1-102 (0.01–0.5 μM) and compared with untreated VSMCs (mock, lower right).

We observed that AngII could suppress the autophagy-related proteins expressions in VSMCs. On the other hand, BP-1-102 treatment could induce autophagy-related proteins expressions in VSMCs, the optional concentration was 0.1 μM. BP-1-102 could maintain the autophagy-related proteins (LC3 and Beclin-1) expressions in VSMCs treated with AngII. Bcl-2 and Bcl-xL are apoptosis related regulators which could also bind to Beclin to inhibit its function. BP-1-102 treatment could reduce apoptosis related protein Bcl-2 expression in VSMCs, and the effects are concentration-dependent. Bcl-xL expression were not reduced by BP-1-102 (Fig. 8a–c). To further observe the effects of BP-1-102 on cells apoptosis. We treated VSMCs with different concentration of BP-1-102 (0.01, 0.1, and 0.5 μM) for 48 h. Annexin 5, which is the indicator of apoptosis, was analyzed with FACS. We observed that BP-1-102 treatment could reduce apoptosis in VSMCs, the optional concentration of BP-1-102 was 0.01 μM. We further found that treatment with BP-1-102 for 48 h significantly reduced AngII-induced cell apoptosis and the optional concentration of BP-1-102 was 0.5 μM (Fig. 8d).

## Discussion

In the current study, for the first time we investigated the protective effect of a novel small molecular STAT3 inhibitor, BP-1-102, on vascular inflammation and AngII-induced AAA progression in ApoE^−/−^ mice. We found that BP-1-102 inhibited inflammatory cells infiltration and suppressed expression of pro-inflammatory cytokines, as well as reduced expression of MMP-2 and MMP-9 in aortic tissues. Moreover, AngII-mediated activation of STAT3 and NF-κB were markedly attenuated in ApoE^−/−^ mice treated with BP-1-102. The protective of BP-1-102 on vascular inflammation and AAA seems to be associated with the inhibition of STAT3 and NF-κB signaling pathways, which are of vital importance in the chronic vascular inflammation during AAA progression. In addition, considering the protective effect of autophagy on AAA and the regulating role of STAT3 on autophagy, we also tested the effect of BP-1-102 on autophagy in ApoE^−/−^ mice and our data revealed that BP-1-102 effectively alleviated AngII-induced impairment of autophagy. Overall, our data proved that the new STAT3 inhibitor, BP-1-102, significantly inhibited AngII-induced AAA progression through reducing vascular inflammation-related JAK2/STAT3 and NF-κB signaling pathways and maintaining autophagy.

JAK2/STAT3 and NF-κB are important pathways mediating various biological processes, including inflammation as well as tissue injury and repair which have been implicated in AAA formation and progression. Ohno et al. analyzed the comprehensive cytokine secretion profiles of 52 cytokines from human AAA using fluorescent beads-based multiplex assay^15^. A high level of JAK2/STAT3 pathway activity in AAA tissue in culture was maintained, which may be attributed to the secretion of endogenous JAK-activating cytokines. These findings suggested that JAK2/STAT3 pathway may play an important role in regulating a subset of cytokines in AAA^15^. NF-κB can promote chronic inflammation in aortic wall and regulate MMPs transcription. NF-κB activities are elevated in human and experimental AAA tissues^11^. Moreover, NF-κB inhibitor has been shown to significantly inhibit the formation of AAA in animal model^14^. Strategies that targeting JAK2/STAT3 and NF-κB showed certain effects on preventing AAA progression. Metformin has been reported to inhibit the AngII-induced AAA progression in mice through decreased the activity of NF-κB and STAT3 signal pathways^29^. Embelin inhibited AAA formation through decreasing IL-6 induced STAT3 and NF-κB inactivation^10^. All these studies indicate that targeting JAK2/STAT3 and NF-κB may be an effective strategy for AAA treatment. A previous study has found that STAT3 inhibitor, S3I-301, reduced the incidence and severity of AngII-induced AAA formation and decreased MMPs activity and the ratio of M1/M2 macrophages^16^. In the present study using AngII-induced AAA model in ApoE^−/−^ mice, BP-1-102 was orally administrated every other day. We evaluated the AAA formation and growth, inflammatory factors expressions and inflammation-related cytokines secretion in AAA tissues at different time points. We found that BP-1-102 could attenuate AAA progression by de-activing the JAK2/STAT3 and NF-κB pathways, maintaining the integrity of aortic wall by reducing expressions of MMPs, and inflammatory factors in AAA tissues. We further investigated whether BP-1-102 could function to suppress the AngII-induced inflammation response in VSMCs in vitro. The results showed that both JAK2/STAT3 and NF-κB activation induced by AngII in VSMCs were suppressed and deactivated by BP-1-102.

Autophagy is responsible for degradation of organelles and long-lived proteins and recycling back the precursor byproducts for biosynthetic processes to maintain cellular homeostasis. Autophagy is impaired with aging and becomes deregulated in cancer, neurodegenerative disease, inflammation and vascular disease^30–32^. Autophagy is an essential process mediating proper vascular function. The association between deregulated autophagy and AAA was investigated and emerging evidence shows that autophagy exhibits protection effect role in AAA progression. Zheng et al. have shown that autophagy gene transcripts ATG4b, Beclin1/ATG6, Bnip3, and Vps34 were all upregulated in AAA tissue^20^. The protective role of autophagy in attenuating the AngII-induced oxidative stress and inflammation have been previously investigated^33^. Assessment of changes in autophagic activity can be performed via transmission electron microscopy detection of autophagosome and autolysosome, processing of cytoplasmic LC3A form to autophagosome-associated LC3B form and the expression of other autophagy-related proteins^34^. In the current study, we intensively observed the autophagy and apoptosis related proteins expressions in VSMCs, and evaluated the effects of BP-1-102 on AngII treated VSMCs. We did not observe a significant change of Bcl-xL but the Bcl-2 expression was suppressed by BP-1-102. Both LC3B and Beclin were upregulated by BP-1-102. Furthermore, we showed that BP-1-102 could induce expression of LC3B, an indicator of autophagy, at the later stage of AAA and reduce apoptosis (Supplementary Fig. 1). These results remind us that BP-1-102 might function to maintain cell protective autophagy during AAA formation. The in vivo protective effect was investigated in AngII-induced experimental AAA model in ApoE^−/−^ mice. LC3B expression was upregulated in AngII^+^ BP-1-102^+^ group, while Bcl-xl but not Bcl-2 was suppressed in AngII^+^BP-1-102^+^ group. These observations suggested that although BP-1-102 could function to maintain autophagy under the circumstances of both in vitro and in vivo, but not through regulating the same Bcl-2 family members.

Recently, accumulating studies tried to find efficient pharmacological treatments of AAA, such as macrolides and tetracyclines, statins, angiotensin converting enzyme inhibitors (ACEI) and angiotensin receptor blockers (ARB), corticosteroids, anti-platelet drugs, mast cell stabilizers all have been studied for AAA treatments; however, the limited effects in clinical trials have been showed^3^. STAT3 inhibitor has been widely studied in the field of anti-tumor treatment. Recently, the role of STAT3 inhibitor in chronic inflammation-related diseases had been investigated. Previous study showed that STAT3 inhibitor, S3I-301, reduced renal fibrosis in a mouse unilateral ureteral obstruction model^35^. Furthermore, the novel STAT3 inhibitor BP-1-102 has been reported to alleviate renal interstitial fibrosis through the inhibition of bone marrow-derived monocyte transition into fibroblast precursors^24^. In cardiovascular diseases, specific STAT3 inhibition has been shown to not only downregulate IL-6-induced collagen synthesis significantly but also effectively regresses the pressure overload-induced cardiac hypertrophy through reducing the fibrosis process^36^. Recent study reported that a STAT3 inhibitor, S3I-301 could reduce AngII-induced AAA formation, and decreased MMPs activity and the ratio of M1/M2 macrophages^16^. These results indicated that AngII-induced AAA formation in mice is dependent on STAT3. However, the role of STAT3 in AAA formation is not fully elucidated. Moreover, it is reported that S3I-201 was neither effective at low concentrations nor orally bioavailable^23^. BP-1-102 was designed as a novel STAT3 inhibitor and is an analog of S3I-201 with much improvement. BP-1-102 has oral bioavailability and could be administrated orally. BP-1-102 functions by directly interacting with STAT3 at a relatively low concentration and has been shown to block the cross talk of JAK2/STAT3 and NF-κB, and regulate autophagy which have been implicated in the molecular mechanism of AAA formation and progression. These advantages of BP-1-102 suggest a promising role in the future clinic application.

In the present study, BP-1-102 was found to suppress JAK2/STAT3 and NF-κB as well as maintain autophagy in VSMCs in a time- and concentration-dependent manner. The results suggest that BP-1-102 may also act as an autophagy regulator in VSMCs. The detailed mechanisms underlying these effects of BP-1-102 on VSMCs remain to be elucidated. There were several limitations to the current study. For example, we only use AngII-induced AAA model in ApoE^−/−^ mice to investigate the effects of BP-1-102. The results generated from the AngII-induced AAA model should be validated in other animal models of AAA. Although we initially confirmed the effects of BP-1-102 on JAK2/STAT3 and NF-κB signaling and autophagy in AAA, the exact molecular mechanism remains largely unknown, further experiments are required.

In conclusion, we proved that orally administration of BP-1-102 could suppress AngII-induced activation of JAK2/STAT3 and NF-κB signaling pathways as well as maintain autophagy, thereby inhibit AngII-induced AAA formation in mice. BP-1-102 may be a promising approach to the treatment of AAA.

## Supplementary information

SUPPLEMENTAL Fig.1
