# Supplementary figures and images for: A novel STAT3 inhibitor attenuates angiotensin II-induced abdominal aortic aneurysm progression in mice through modulating vascular inflammation and autophagy
Source: Cell Death Dis. 2020 Feb 18;11(2):131. doi: 10.1038/s41419-020-2326-2 (PMC7028955; doi:10.1038/s41419-020-2326-2)

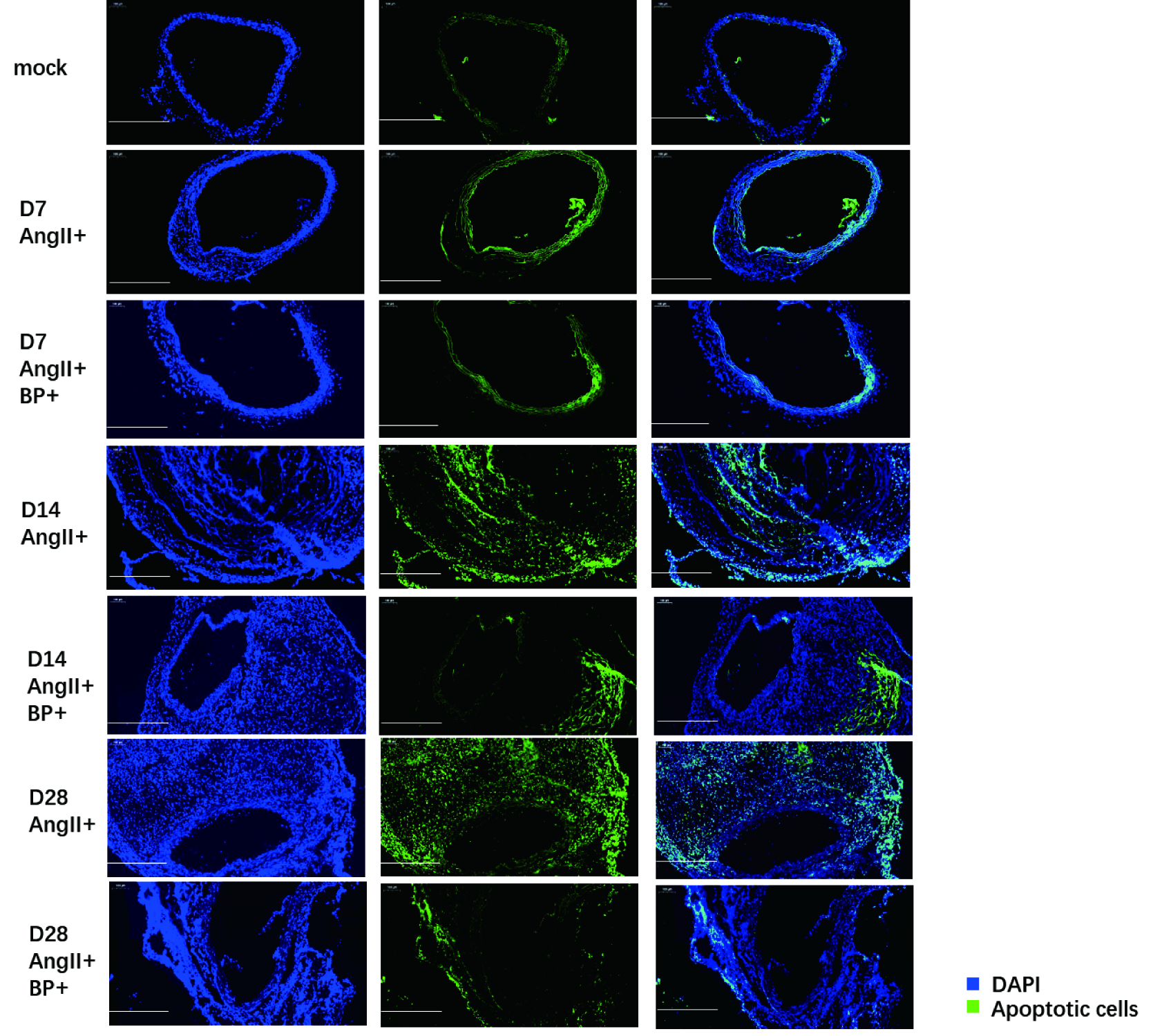

Supplement: Supplementary file 1 — SUPPLEMENTAL Fig.1 [file 41419_2020_2326_MOESM1_ESM.tif]
